# Utility of the ACTH/cortisol ratio for differentiating pituitary microadenomas from macroadenomas in dogs with pituitary-dependent hypercortisolism

**DOI:** 10.1093/jvimsj/aalag269

**Published:** 2026-10-29

**Authors:** George Stylianou, Federico Vassallo, Naohito Nishii, Alessia Diana, Antonio M Tardo, Francesca Del Baldo, Federico Fracassi

**Affiliations:** Small Animal Practice, Paphos 8300, Cyprus; Animal House Veterinary Clinic, Capoterra 09012, Italy; Joint Department of Veterinary Medicine, Faculty of Applied Biological Science, Gifu University, Gifu 501-1193, Japan; Department of Veterinary Medical Science, University of Bologna, Ozzano dell’Emilia, Bologna 40064, Italy; Department of Veterinary Medical Science, University of Bologna, Ozzano dell’Emilia, Bologna 40064, Italy; Department of Veterinary Medical Science, University of Bologna, Ozzano dell’Emilia, Bologna 40064, Italy; Department of Veterinary Medical Science, University of Bologna, Ozzano dell’Emilia, Bologna 40064, Italy

**Keywords:** Cushing’s disease, pituitary-dependent hypercortisolism, pituitary adenoma, ACTH/cortisol ratio, canine, biomarker

## Abstract

**Background:**

No simple, blood-based biomarker to estimate tumor size in dogs with pituitary-dependent hypercortisolism (PDH) has been evaluated.

**Hypothesis/Objectives:**

Determine whether the adrenocorticotropic hormone (ACTH)/cortisol ratio differentiates pituitary microadenomas from macroadenomas in dogs with PDH and correlates with pituitary size.

**Animals:**

Seventy privately-owned dogs with PDH (microadenomas, *n* = 30; macroadenomas, *n* = 40).

**Methods:**

Retrospective multicenter observational study. Endogenous ACTH and cortisol concentrations obtained at the same time point before treatment were used to calculate the ACTH/cortisol ratio. Pituitary tumor size was classified using the pituitary-to-brain (P/B) ratio. Diagnostic performance was evaluated using receiver operating characteristic analysis and multivariable logistic regression.

**Results:**

Dogs with macroadenomas had significantly higher ACTH/cortisol ratios than dogs with microadenomas (median, 9.12; IQR, 6.46-14.28 vs median, 4.90; IQR, 3.08-8.10; *P* < .001). The ratio showed moderate discrimination for differentiating macroadenomas from microadenomas with an area under the curve of 0.77 (95% CI, 0.66-0.89; *P* < .001). A cutoff >5.69 yielded 85% sensitivity and 60% specificity (positive likelihood ratio 2.13), whereas a higher ratio >11.74 achieved 90% specificity and 35% sensitivity (positive likelihood ratio 3.50). The ACTH/cortisol ratio showed moderate positive correlation with pituitary size (Spearman’s ρ = 0.48; 95% CI, 0.26-0.64; *P* < .001) and remained independently associated with macroadenoma classification in multivariable analysis (OR, 1.19; 95% CI, 1.05-1.40).

**Conclusions and clinical importance:**

The ACTH/cortisol ratio is associated with pituitary tumor size in dogs with PDH and may support clinical risk stratification, particularly when advanced imaging is unavailable.

## Introduction

Pituitary-dependent hypercortisolism (PDH) in dogs is caused by adrenocorticotropic hormone (ACTH)-secreting pituitary adenomas (ACTH-dependent hypercortisolism^1^^,^^2^), which are classified as microadenomas or macroadenomas based on tumor size.^3^ Differentiating between these tumors is clinically relevant because macroadenomas are more likely to invade surrounding structures, thereby influencing prognosis and therapeutic strategies.^4^ Although computed tomography (CT) and magnetic resonance imaging (MRI) are the reference methods for differentiating between these tumor classes and provide detailed diagnostic information on tumor size,^5–7^ they are not routinely performed in all dogs with PDH. Therefore, a simple, low-cost, blood-based biomarker that could assist in estimating pituitary tumor size could facilitate clinical decision-making and prioritization of diagnostic and therapeutic resources.

Macroadenomas may produce disproportionately high ACTH concentrations relative to cortisol output,^8–12^ reflecting impaired glucocorticoid negative feedback.^6^ This pathophysiological mechanism provides the rationale for evaluating the ACTH/cortisol ratio as a potential discriminator between macro- and microadenomas.

In Cushing’s disease in humans, higher ACTH/cortisol ratios have been associated with larger tumors and worse prognosis.^13^ However, this ratio has not been evaluated as a discriminator between pituitary microadenomas and macroadenomas in veterinary medicine.

Our aim was to determine whether the ACTH/cortisol ratio can differentiate pituitary microadenomas from macroadenomas in dogs with PDH and whether it correlates with pituitary size. We hypothesized that the ratio would have diagnostic utility in distinguishing between tumor size categories and would be positively correlated with pituitary size.

## Materials and methods

### Animals and study design

Our retrospective multicenter observational study included 43 and 27 privately-owned dogs with PDH from the digital databases of the University of Bologna Veterinary Teaching Hospital, Italy and the Laboratory of the Animal Medical Center, Gifu University, Japan, respectively.

Dogs were eligible for inclusion if they were diagnosed with PDH, had undergone head CT within three months of blood sampling, and had endogenous ACTH and cortisol concentrations measured from the same sampling time before treatment using trilostane, mitotane, or cabergoline, or before hypophysectomy.

Hypercortisolism was diagnosed based on the presence of at least two compatible clinical signs (polyuria, polydipsia, polyphagia, abdominal enlargement, thin skin, hyperpigmentation, calcinosis cutis, alopecia, polypnea) together with at least one of the following endocrine test results: post-ACTH stimulation cortisol concentration >22 μg/dL^14^ or 8-hour cortisol concentration after low-dose dexamethasone suppression test (LDDST) >1.2 μg/dl.^14^

Detailed descriptions of sample collection and handling, endocrine testing, and pituitary tumor classification are provided in Appendix.

### Statistical analysis

Statistical analyses were performed using GraphPad Prism 11 (GraphPad Software, San Diego, CA) and R (version 4.5.2; R Foundation for Statistical Computing, Vienna, Austria) in RStudio (version 2026.01.0 + 392; Posit Software, PBC, Boston, MA, United States). Continuous and categorical variables were compared using Mann–Whitney *U* and Fisher’s exact tests, respectively. Age and body weight were compared between institutions using Welch’s *t*-test. Effect sizes were expressed as Hodges–Lehmann median differences and rank-biserial correlations. Associations with the pituitary-to-brain (P/B) ratio were assessed using Spearman’s correlation.

A linear regression model derived from dogs with microadenomas was used to calculate residual ACTH results, which were compared between groups using the Mann–Whitney *U* test. Diagnostic performance was assessed by receiver operating characteristic (ROC) analysis, with optimal cutoffs determined using the Youden index. Correlated ROC curves were compared using DeLong’s method. Multivariable logistic regression including endogenous ACTH concentration and the ACTH/cortisol ratio was used to evaluate independent associations with macroadenoma classification. Collinearity was assessed using Pearson correlation and variance inflation factors (VIFs). Model discrimination and calibration were evaluated by area under the curve (AUC) and the Hosmer–Lemeshow test, respectively. Statistical significance was set at *P* < .05.

## Results

### Study population

Seventy dogs with PDH were included and classified as having microadenomas (*n* = 30) or macroadenomas (*n* = 40) based on the P/B. The median P/B was 0.24 (range, 0.14-0.31) in dogs with microadenomas and 0.56 (range, 0.34-1.59) in dogs with macroadenomas. Baseline characteristics were similar between groups (Table 1). No significant differences in age, body weight, or sex distribution were observed between dogs with microadenomas and macroadenomas. Comparison of the study populations from the two institutions showed no significant difference in age (*P* = .65). However, dogs from the University of Bologna had a significantly higher body weight than dogs from Gifu University (15.96 ± 10.03 kg vs 7.71 ± 3.78 kg, *P* < .001). No significant differences were observed between institutions in P/B, endogenous ACTH concentration, or serum cortisol concentration (*P* = .31, *P* = .20, and *P* = .07, respectively).

**Table 1:** Baseline characteristics and endocrine variables of dogs with pituitary-dependent hypercortisolism classified by pituitary tumor size.

**Variable**	**Microadenoma (*n* = 30)**	**Macroadenoma (*n* = 40)**	***P* value**
**Age (years)**	10.65 (7.9-12.9)	10.0 (8.0-11.9)	.53
**Body weight (kg)**	10.5 (5.8-13.45)	10.7 (6.58-17.78)	.43
**Male sex**	12/30 (40%)	21/40 (52%)	.34
**Endogenous ACTH (pg/mL)**	20.5 (13.0-29.5)	46.0 (30.0-61.5)	<.001
**Cortisol (μg/dL)**	4.07 (3.05-6.91)	4.18 (3.05-6.55)	.86
**ACTH/cortisol ratio**	4.9 (3.08-8.10)	9.12 (6.46-14.28)	<.001
**P/B ratio**	0.24 (0.14-0.31)	0.56 (0.34-1.59)	NA

Data are presented as median (interquartile range) unless stated otherwise; P/B ratio is presented as median (range). Continuous variables were compared using Mann–Whitney *U* test and categorical variables using Fisher’s exact test. P/B ratio was used for tumor classification and was not compared statistically between groups.

Abbreviation: NA = not applicable.

### Group comparisons

Dogs with macroadenomas had significantly higher ACTH/cortisol ratio compared with dogs with microadenomas (median, 9.12; IQR, 6.46-14.28; *n* = 40 vs median, 4.90; IQR, 3.08-8.10; *n* = 30; *P* < .001), with a Hodges–Lehmann median difference of 4.10 (rank-biserial correlation r = 0.55). Endogenous ACTH concentrations were significantly higher in macroadenomas compared with microadenomas (median, 46.00; IQR, 30-61.5 pg/mL; *n* = 40 vs median, 20.5; IQR, 13-29.5 pg/mL; *n* = 30; *P* < .001), with a Hodges–Lehmann median difference of 19.25 pg/mL (rank-biserial correlation r = 0.51). Serum cortisol concentrations did not differ significantly between dogs with microadenomas and macroadenomas (median, 4.07; IQR, 3.05-6.91 μg/dL vs median, 4.18; IQR, 3.05-6.55 μg/dL; *P* = .86). The distributions of ACTH/cortisol ratio and endogenous ACTH are shown in Figures 1 and 2.

**Figure 1:**
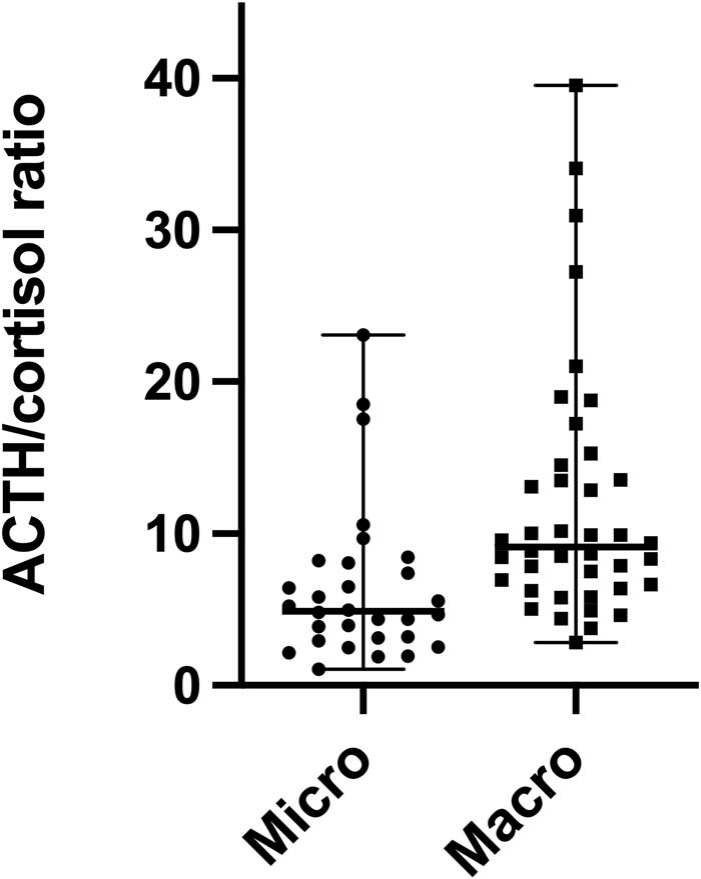
Distribution of ACTH/cortisol ratio in dogs with pituitary microadenomas (*n* = 30) and macroadenomas (*n* = 40). The horizontal lines represent the median, minimum, and maximum value of each group. Individual points represent individual dogs.

**Figure 2:**
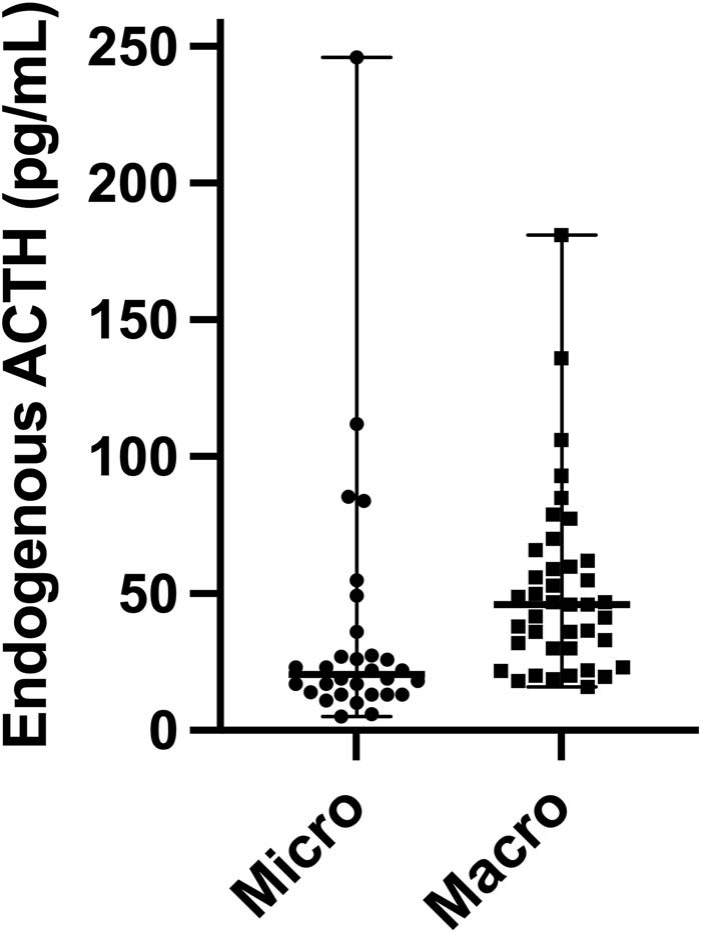
Distribution of endogenous ACTH in dogs with pituitary microadenomas (*n* = 30) and macroadenomas (*n* = 40). The horizontal lines represent the median, minimum, and maximum value of each group. Individual points represent individual dogs.

### Correlation with pituitary size

Both ACTH/cortisol ratio and endogenous ACTH concentration showed moderate positive correlations with P/B (Spearman’s ρ = 0.48; 95% CI, 0.26-0.64; *P* < .001 and ρ = 0.41; 95% CI, 0.19-0.60; *P* < .001, respectively; *n* = 70; Figure 3).

**Figure 3:**
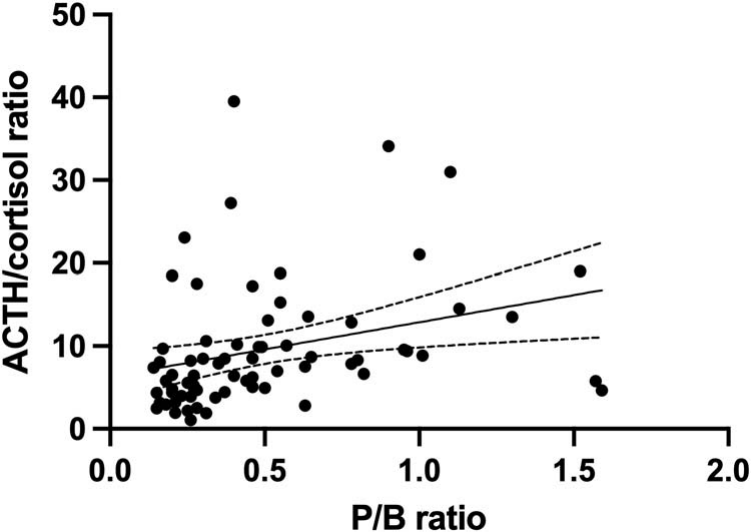
Relationship between ACTH/cortisol ratio and pituitary-to-brain (P/B) ratio in dogs with PDH (*n* = 70). Each point represents an individual dog. The solid line represents the linear regression fit for visual representation, with 95% confidence intervals. Correlation was assessed using Spearman’s rank correlation.

### Diagnostic discrimination

Receiver operating characteristic analysis showed that the ACTH/cortisol ratio provided moderate discrimination between macroadenomas and microadenomas, with an AUC of 0.77 (95% CI, 0.66-0.89; *P* < .001). The Youden-optimal cutoff (>5.69) yielded a sensitivity of 85% and specificity of 60%, corresponding to a positive likelihood ratio (LR+) of 2.13. To establish a clinically useful rule-in threshold for macroadenoma classification, a higher cutoff (>11.74) was selected, achieving specificity of 90% and sensitivity of 35%, with a LR+ of 3.50. Endogenous ACTH concentration showed similar discriminatory performance, with an AUC of 0.76 (95% CI, 0.63-0.88; *P* < .001; Figure 4). The Youden-optimal cutoff (>27.15 pg/mL) yielded a sensitivity of 78% and specificity of 73%, corresponding to a LR+ of 2.9. For rule-in classification of macroadenomas, a higher threshold (>84.5 pg/mL) increased specificity to 90% but decreased sensitivity (13%), resulting in a LR+ of 1.25. Comparison of correlated ROC curves (ACTH/cortisol and endogenous ACTH) using DeLong’s method showed no significant difference between AUCs (Z = 0.31; *P* = .75; 95% CI for AUC difference, −0.087 to 0.121).

**Figure 4:**
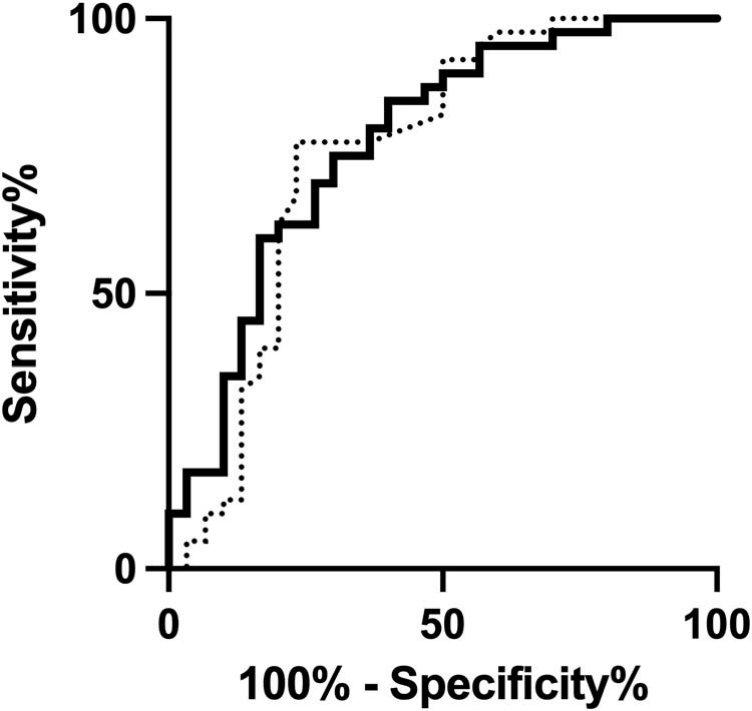
Receiver operating characteristic curves for the use of ACTH/cortisol ratio and endogenous ACTH to differentiate pituitary microadenomas from macroadenomas in dogs with PDH. The solid line represents the ACTH/cortisol ratio and the dotted line represents endogenous ACTH.

### Residual ACTH model

Residual ACTH results, derived from the microadenoma-based regression model, were significantly higher in dogs with macroadenomas compared with microadenomas (median, 12.28 pg/mL vs median, −5.42 pg/mL; *P* < .001; Figure 5). The Hodges–Lehmann median difference was 18.68 pg/mL, consistent with a relative excess of ACTH secretion for a given cortisol concentration in macroadenomas. The rank-biserial correlation was 0.48, indicating a moderate-to-large effect size.

**Figure 5:**
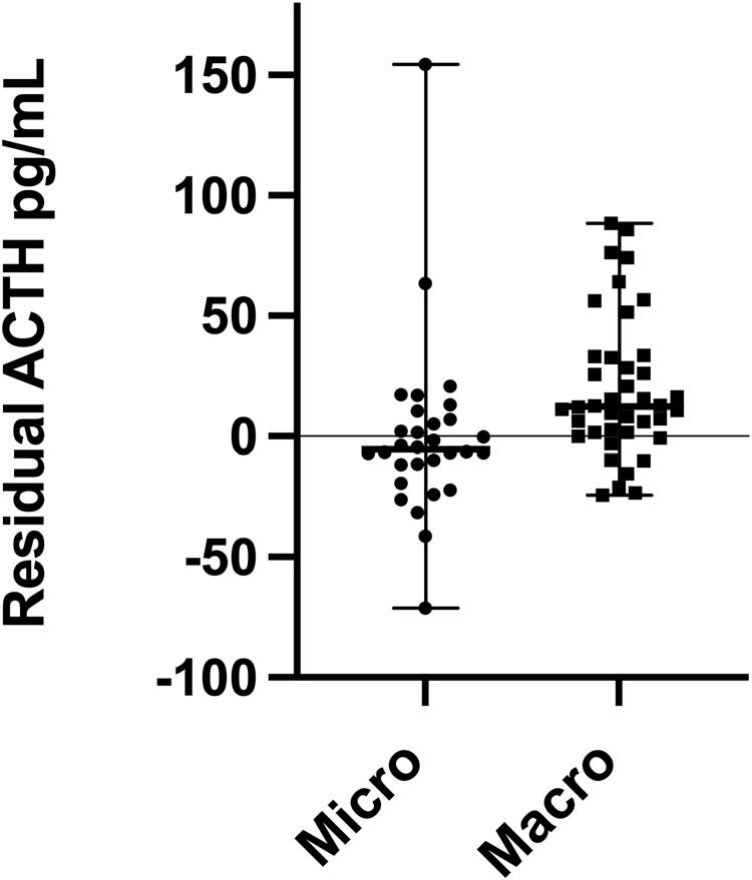
Distribution of residual ACTH values in dogs with pituitary microadenomas (*n* = 30) and macroadenomas (*n* = 40). The horizontal lines represent the median, minimum, and maximum value of each group. Individual points represent individual dogs.

### Multivariable logistic regression

Endogenous ACTH concentration and the ACTH/cortisol ratio were moderately correlated (Pearson r = 0.54; 95% CI, 0.34-0.68; *P* < .001), with approximately 29% shared variance between predictors (r^2^ = 0.29). Variance inflation factor was 1.40, indicating absence of problematic multicollinearity. In multivariable logistic regression including both endogenous ACTH concentration and the ACTH/cortisol ratio (*n* = 70), only the ratio remained independently associated with macroadenoma classification (OR, 1.19 per unit increase; 95% CI, 1.05-1.40), whereas ACTH was not significant (OR, 0.997; 95% CI, 0.98-1.02). The model showed moderate discriminatory ability with an AUC of 0.77 (95% CI, 0.65-0.88; *P* < .001). Model calibration was adequate, with no evidence of lack-of-fit based on the Hosmer–Lemeshow test (χ^2^ = 7.66; *P* = .47).

## Discussion

We demonstrated that the ACTH/cortisol ratio is associated with pituitary tumor size in dogs with PDH and provides moderate discrimination between microadenomas and macroadenomas. The ratio was positively correlated with quantitative measures of pituitary size (P/B) and remained independently associated with macroadenoma classification in multivariable analysis. Together, these findings suggest that the ratio captures clinically relevant information beyond ACTH concentration alone, although the incremental diagnostic gain appears modest.

Receiver operating characteristic analysis indicated moderate diagnostic performance for both the ACTH/cortisol ratio (AUC, 0.77) and endogenous ACTH concentration (AUC, 0.76), indicating comparable discrimination between tumor size categories. Although the difference in AUCs was small, multivariable modeling suggests that the ACTH/cortisol ratio may provide additional predictive information beyond endogenous ACTH concentration. Each unit increase in the ratio was associated with increased odds of macroadenoma classification, supporting its potential utility as a biomarker of tumor size. Furthermore, both the ratio and endogenous ACTH showed moderate positive correlations with P/B, reinforcing the biological plausibility of the observed associations.

An important methodological consideration is the definition of pituitary macroadenoma. Historically, size-based classifications in dogs were largely derived from necropsy observations,^15^^,^^16^ in which pituitary tumor dimensions were assessed and subsequently extrapolated to in vivo imaging studies. This approach led to the use of absolute size thresholds, including the 1 cm cutoff derived from human medicine.^12^^,^^17^ However, this cutoff may be inappropriate in dogs given variability in brain size across breeds and may result in misclassification. Relative measurements such as P/B,^18^^,^^19^ account for this variability by normalizing pituitary size to brain dimensions. In our study, tumor classification was based on P/B, which provides a size assessment independent of body size. Although P/B is subject to measurement variability and lacks universal standardization, these limitations are not unique and similarly affect absolute size measurements, including pituitary height. The pituitary-brain ratio normalizes pituitary size to brain dimensions and was also associated with endocrine markers in our study, suggesting that it reflects functionally relevant differences in tumor size beyond absolute measurements.

From a mechanistic perspective, the ACTH/cortisol ratio may better reflect the functional dynamics of the hypothalamic-pituitary-adrenal (HPA) axis feedback than ACTH concentration alone.^13^^,^^20–23^ Higher results may indicate relative ACTH excess despite increased cortisol concentrations,^8^^,^^13^^,^^24^^,^^25^ consistent with impaired feedback sensitivity in larger tumors. Although ACTH represents pituitary secretion at a single time point, the ratio incorporates the downstream cortisol response and therefore contextualizes ACTH relative to glucocorticoid output. The ratio was chosen because ACTH secretion is characterized by substantial biological variability, reflecting pulsatile hypothalamic-pituitary activity and tumor-related dysregulation, whereas cortisol represents the downstream integrated adrenal response. This distinction has been described in dogs with PDH, in which ACTH demonstrates dynamic secretion patterns, whereas cortisol reflects the resultant adrenal output.^26^ Macroadenomas may exhibit impaired glucocorticoid negative feedback, leading to disproportionate ACTH secretion relative to circulating cortisol concentrations.^8^^,^^13^^,^^24^^,^^25^ Consistent with this assumption, cortisol concentrations did not differ between dogs with microadenomas and those with macroadenomas in our study, suggesting that the discriminatory ability of the ACTH/cortisol ratio primarily reflects differences in ACTH secretion rather than differences in cortisol output. In this context, the independent association of the ratio in multivariable analysis supports the hypothesis that it reflects altered feedback dynamics rather than increased ACTH concentrations alone. Taken together, these findings suggest that the ACTH/cortisol ratio may represent a simple physiologic index of pituitary ACTH secretion relative to glucocorticoid output, providing complementary information to endogenous ACTH concentration.

Additional support for this interpretation is provided by the residual ACTH model. Using the microadenoma group as a biological reference, regression modeling defined the expected ACTH response relative to cortisol concentrations under presumed preserved feedback conditions. Microadenomas were selected as the reference group because they generally are considered to maintain a more physiologically regulated HPA feedback relationship compared with larger tumors.^18^^,^^27^ Dogs with macroadenomas demonstrated a marked upward deviation from this reference relationship, with substantially higher residual endogenous ACTH concentrations. This finding indicates relative ACTH excess when interpreted within the context of cortisol concentrations instead of absolute hypersecretion alone. The magnitude of this difference was large, supporting the biological relevance of the observation. Together, the persistence of the ratio as an independent predictor and the positive residual shift in macroadenomas support the hypothesis of altered physiological ACTH-cortisol coupling in larger tumors.

From a clinical standpoint, the Youden-optimal cutoff for the ACTH/cortisol ratio (>5.69) provided balanced sensitivity and specificity, whereas a higher threshold (>11.74) achieved high specificity and may support identification of dogs more likely to have macroadenomas. The corresponding positive likelihood ratios indicate that the higher cutoff of the ACTH/cortisol ratio provides a moderate increase in the post-test probability of macroadenoma classification, whereas endogenous ACTH concentration showed limited rule-in performance at similar specificity. Although the ratio cannot replace advanced imaging, it may assist in prioritizing further advanced diagnostics or informing clinical discussions regarding prognosis and treatment planning. The ACTH/cortisol ratio could function as an initial triage biomarker to help identify dogs in which advanced imaging may be particularly warranted for assessment of pituitary tumor size and treatment planning.

It is important to acknowledge that the ACTH/cortisol ratio mathematically incorporates ACTH, potentially introducing collinearity in multivariable modeling. However, regression diagnostics did not indicate instability of the model, and collinearity between ACTH and the ratio was low. These findings suggest that the independent association of the ratio reflects meaningful physiological variability rather than purely mathematical coupling. Nevertheless, the overall diagnostic performance observed in our study was moderate and the ratio should be considered an adjunct biomarker instead of a standalone diagnostic tool.

Our study had some limitations. First, the retrospective design introduced potential selection bias, and in some dogs, CT imaging was performed up to three months after hormone sampling. Data were obtained from 2 institutions, and although validated chemiluminescent assays were used, inter-laboratory variability cannot be completely excluded. Center was not formally included as a covariate in multivariable modeling. Therefore, potential clustering effects cannot be entirely ruled out. However, the same endocrine assays and similar diagnostic protocols were used at both institutions, decreasing the likelihood of center-related bias. In addition, pituitary tumor size was classified using P/B. Although this approach is widely accepted and accounts for differences in brain size among dogs, imaging-based morphometric measurements are inherently subject to intra- and interobserver variability. Consequently, measurements close to the diagnostic threshold may be susceptible to minor classification differences. Moreover, physiological (stressful events) or pathological conditions (concurrent systemic illness) that can cause hypercortisolemia were not specifically evaluated in our study. Such states could alter the ACTH/cortisol ratio by increasing cortisol concentrations and potentially decreasing the ratio’s discriminatory ability. Although sample size was substantial, further validation in larger prospective cohorts is needed. Additionally, the residual ACTH model was exploratory and requires independent confirmation.

In conclusion, the ACTH/cortisol ratio represents a simple, inexpensive biomarker associated with pituitary tumor size in dogs with PDH. Its independent association with macroadenoma classification and its correlation with pituitary size support its potential role as an adjunct biomarker to aid clinical risk stratification in dogs with PDH.

## Off-label antimicrobial declaration

Authors declare no off-label use of antimicrobials.

## Institutional animal care and use committee or other approval declaration

Authors declare no institutional animal care and use committee or other approval was needed.

## Appendix: Sample collection, endocrine tests and pituitary tumor classification

For the ACTH stimulation test (ACTHST), blood samples were collected, at both centers, before and 60 minutes after administration of synthetic tetracosactide.^28^ At the University of Bologna, Cosacthen (Dechra Veterinary Products S.r.l., The Netherlands) was administered IV (5 μg/kg), whereas at Gifu University, Cortrosyn (Alfresa Corporation, Tokyo, Japan) was administered IM (250 μg/dog >10 kg or 125 μg/dog <10 kg).^29^^,^^30^ For the low-dose dexamethasone suppression test (LDDST), blood samples were collected before, and at 4 and 8 hours after IV administration of dexamethasone (0.01 mg/kg).^31^ Blood samples for cortisol measurement were collected in serum separating tubes. Coagulated blood samples were centrifuged for 10 minutes at 3000 *g*; serum was immediately transferred to plastic tubes, stored at 4 °C and analyzed the same day or stored at *-*20 °C and thawed immediately before analysis. Blood samples for the determination of the endogenous ACTH concentration were collected into EDTA-coated plastic tubes and placed on ice. The samples were immediately centrifuged at 4 °C, 500 *g* for 8 minutes, and the plasma was immediately transferred to plastic tubes, stored at 4 °C and analyzed within 8 hours or stored at *-*80 °C and thawed immediately before analysis.

Serum cortisol and endogenous ACTH concentrations were measured at both centers using separate chemiluminescent enzyme immunoassays (Immulite 2000, Siemens Healthcare), which have been validated for use in dogs.^32^^,^^33^

The ACTH/cortisol ratio was calculated as ACTH (pg/mL) divided by cortisol (μg/dL) and expressed as a unitless index.

Pituitary tumors were classified as microadenomas (P/B *≤* 0.31) or macroadenomas (P/B > 0.31) according to P/B.^3^^,^^18^

## Abbreviations

ACTH: adrenocorticotropic hormone
ACTHST: ACTH stimulation test
AUC: area under the curve
CI: confidence interval
CT: computed tomography
HPA: hypothalamic-pituitary-adrenal axis
IM: intramuscular
IQR: interquartile range
IV: intravenous
LDDST: low-dose dexamethasone suppression test
LR+: positive likelihood ratio
MRI: magnetic resonance imaging
OR: odds ratio
P/B: pituitary-to-brain ratio
PDH: pituitary-dependent hypercortisolism
ROC: receiver operating characteristic
VIF: variance inflation factor
